# Total colectomy in Vascular Ehlers Danlos syndrome a case report and literature review

**DOI:** 10.1016/j.amsu.2021.102948

**Published:** 2021-10-15

**Authors:** Khalid Elhattabi, Hasna Benghait, Abdelilah Elbakouri, Mounir Bouali, Fatimazahra Bensardi, Abdelaziz Fadil

**Affiliations:** a Departement of General Surgery, University Hospital Centre Ibn Rochd, Casablanca, Morocco; b Faculty of Medecine and Pharmacy, Hassan II University, Casablanca, Morocco

**Keywords:** Ehlers-danlos syndrom, Colonic perforation, Total colectomy

## Abstract

**Introduction:**

Ehlers Danlos syndromes (EDS) are a group of genetic disorders, characterized by skin hyperelasticity, joint hyperlaxity and tissue weakness. Vascular EDS is rare and is differs from other types of EDS by an inconsistent acrogenic morphotype and the occurrence of severe digestive and vascular complications, which can be lifethreatening.

**Case presentation:**

We report the case of a 27-year-old man with a type IV vascular Ehlers-Danlos syndrome revealed by a colonic perforation after appendectomy for peritonitis secondary to appendicitis. The etiology of the perforation remained a challenge till a genetic research was carried out for COL3A1 gene mutation, which was positive in favor of vascular Ehlers Danlos disease. Then, a totalization of the colectomy with ileorectal anastomosis was performed.

**Discussion:**

Vascular Ehlers Danlos syndrome (VEDS) is due to qualitative and quantitative abnormalities in the synthesis of type III collagen, which is a major constituent of the vessel wall, skin, joint capsules, uterus and gastrointestinal tract, particularly the colon. Colonic perforation, particularly sigmoidal perforation, is the most frequent complication in SEDV and most often precedes the molecular diagnosis. Colonic perforations are uncommon. The Hartmann procedure is a well-established surgical treatment modality, especially for emergency surgery. Given the iterative risk of colonic perforation and anastomotic leakage, preventive treatment by total colectomy with ileo-rectal anastomosis or definitive ileostomy is recommended by several authors.

**Conclusion:**

SEDV is a rare pathology with a difficult diagnosis. However, it should be keeped in mind when there is any spontaneous colonic perforation in the young people.

## Highlights

• Ehlers danlos syndromes are a group of genetic disorders of the connective tissue.
• Life expectancy is reduced due to spontaneous vascular rupture or colonic perforation.
• Colonic perforations are uncommon. They occur in the context of diverticulitis in adults.
• The diagnosis is difficult because of the rarity of the pathology and the inaccessibility of genetic tests.
• The surgical management of colonic perforations in ehlers danlos syndromes is not codified.

## 1 Introduction

Ehlers Danlos syndromes (EDS) are a group of genetic disorders of the connective tissues, characterized by skin hyperelasticity, joint hyperlaxity and tissue weakness [1].

Vascular EDS is rare and differs from other types by an inconsistent acrogenic morphotype and the occurrence of severe digestive and vascular complications, which can be life-threatening. Its prevalence is estimated at 1 per 150 000 [2].

Life expectancy is reduced (mean age 45–50 years), due to spontaneous vascular rupture or colonic perforation [3]. The first complication may occur before the twentieth year in 25% of cases and 80% have a risk of complications before the fourteenth year [3].

We report the case of a 27 year old man with vascular Ehlers Danlos syndrome revealed by sigmoid perforation for whom a total colectomy was performed with deferred ileorectal anastomosis as a preventive measure. The aim of this case is to discuss the place of preventive total colectomy in Ehlers Danlos disease according to literature review. This case have been reported in line with scare guideline 2020 [4].

### 1.1 Case report

A 27-year-old man with history of appendectomy and peritonitis due to sigmoid perforation. A segmental sigmoidal resection with Hartmann colostomy were performed. The specimen analysis did not reveal any inflammatory sign of bowel disease.

Three months later, the patient presented an occlusive syndrome complicated with stoma and the small bowel necrosis. A 43-cm small bowel resection with end to end anastomosis and 10 cm colonic resection associated with a left iliac fossa colostomy rebuilt were performed. The specimen analysis showed moderate congestive interstitial colitis with the presence of numerous eosinophilic polynuclear cells exceeding 40 PNE/field, which could be in favor of eosinophilic colitis, without granulomatous lesions or other signs of IBD. Examination of the bowel resection showed a regular moderately inflammatory mucosa without cryptic abnormalities with some giant cells and rare granulomas in the muscular.

**Fig. 1:**
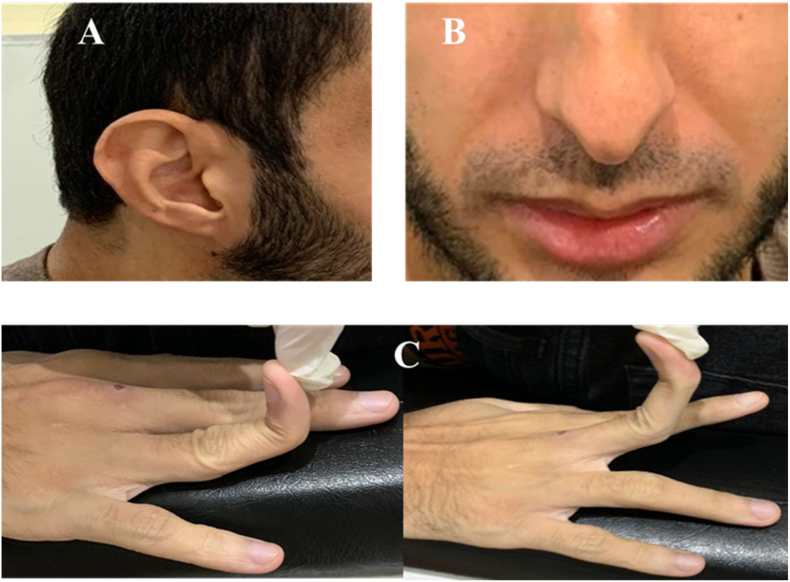
*Morphotypic aspects in favor of Vascular Ehlers Danlos syndrome encountered in our patient.* A. Hypoplasia of the ear lobule; B. horizontal and slightly hemmed lips, pinched nose. C. Hyperlaxity of the small joints.

On third hospitalization, physical examination showed (Fig. 1):

- Thin, loose skin,
- Hypoplasia of the ear lobule [A].
- Madonna face appearance: pinched nose, horizontal lips, prominent cheekbones, and sunken and dark circled eyes [B].
- Acrogeria of the hands [C].

Abdominal examination showed:

- Atrophic aspect of the median laparotomy scar
- Postoperative eventration of the linea alba with a ring sac 10 cm in diameter.

The remain of physical examination was normal.

**Fig. 2:**
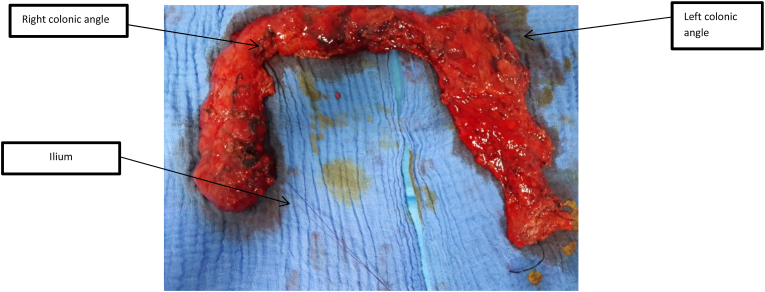
Image of the surgical specimen of the totalization of the colectomy.

**Fig. 3:**
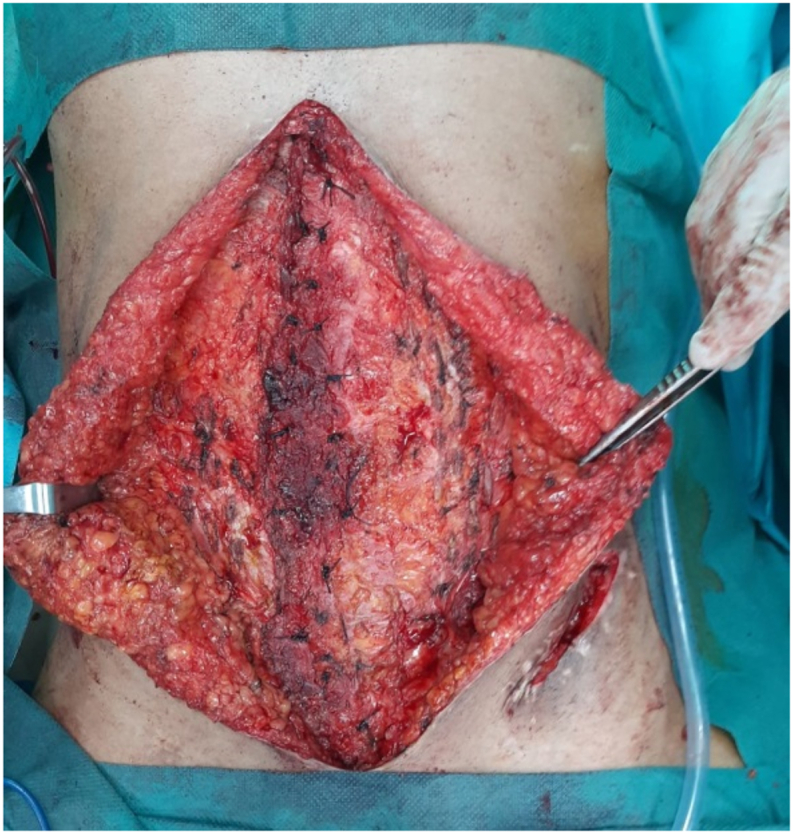
Intraoperative image of the cure of the white line eventration by paletot.

The etiology of the perforation remained a challenge for this patient and after morphotype analysis, a genetic research was carried out for COL3A1 gene mutation, which was positive in favor of vascular Ehlers Danlos disease. Then, a totalization of the colectomy with ileorectal anastomosis was performed (Fig. 2), associated with incisional hernia treatment by Paletot technique (Fig. 3). The postoperative and long term follow-up was uneventfull.

## 2 Discussion

Vascular Ehlers Danlos syndrome (VEDS) is due to qualitative and quantitative abnormalities in the synthesis of type III collagen, which is the major component of the vessel wall, skin, joint capsules, uterus and gastrointestinal tract, particularly the colon [5]. This disease is related to mutations of the COL3A1 gene (exceptionally COL1A1), located in chromosome 2 at the q24.3 - q31 locus (OMIM 130 050), coding for the pro-α(1) chain of type III collagen **(**5)**,** [6]. It is transmitted according to an autosomal dominant mode, however the rate of neomutation remains high (50%), explaining the sporadic cases encountered. This was the case in our observation [6,7].

The diagnosis is clinically established with the help of clinical criteria summarized by the Villefranche (1997) and more recently the New York (2017) classifications (Table 1) [8]. Only genetic study confirms the diagnosis by demonstrating the heterozygous mutation of the COL3A1 gene. For intestines complications, Colonic perforations are uncommon. They occur in the context of diverticulitis in adults over 40 years of age or abdominal trauma. The sigmoid perforation is the most frequent complication in SEDV often before the molecular diagnosis.

**Table 1:** Diagnostic criteria reported in the international classification of (2017) [8].

Diagnostic criteria
**Major criteria** Family history of vascular SED with a causal COL3A1 variant Spontaneous arterial rupture in young adults Spontaneous sigmoidal perforation in the absence of diverticular pathology or other known intestinal pathology Uterine rupture during the third trimester of pregnancy in the absence of a history of cesarean section and/or severe perineal tear peripartum. Fistula between the cavernous sinus and the carotid artery in the absence of trauma.
**Minor criteria** Contusions not related to a known trauma and/or on unusual sites such as the back and cheek Thin, transparent skin with a more visible venous network Characteristic facial appearance Spontaneous pneumothorax Acrogeria Equine varus foot Congenital dislocation of the hips Hyperlaxity of small joints Rupture of tendons and muscles Keratoconus Retraction and fragility of the gums Early onset of varicose veins [women under 30, nulliparous].
The diagnosis suggesting a vascular type requires at a minimum: A family history of vascular EDS Arterial rupture or dissection in individuals under 40 years of age Unexplained sigmoid rupture or spontaneous pneumothorax in the presence of other signs consistent with vascular SED.

By the way, any idiopathic colonic perforation in a young adult, in the absence of colonic pathology, should require a genetic study for SEDV [9]. In 47% of cases, patients with a colonic perforation are at risk of early recidivisme within 20 months of the first perforation in the absence of adequate therapeutic management [2].

In the literature, the surgical management of colonic perforations in SEDV is not codified, the main reasons being the rarity of the disease, the absence of a formal diagnosis at the time of the first colonic perforation, and the need for emergency surgical treatment [10,11].

The Hartmann procedure is a well-established surgical treatment modality, especially for emergency surgery. Given the iterative risk of colonic perforation and anastomotic leakage, preventive treatment by total colectomy with ileo-rectal anastomosis or definitive ileostomy is recommended by several authors [8]. However, the risk of ascendant, transverse, descendant and rectal perforation is rare [11].

A recently published meta-analysis by Speacke et al. describes 109 operations performed in 51 patients with EDS reported in 44 case series and allows to establish a management strategy in EDS by evaluating the risk of intestinal perforation recidivisme in the therapeutic management. Indeed, the rate of recidivisme is significantly higher after colo-colic anastomosis, compared to ileo-rectal anastomosis (10/8, 55% vs 1/14, 7% respectively), survival analysis also increases with total colectomy compared to other operative modes. The author therefore suggests that total colectomy with ileorectal anastomosis or definitive ileostomy should be performed to prevent the subsequent risk of perforation in any patient with confirmed Ehlers Danlos syndrome.

This strategy would limit the risk of certain immediate complications (suture loosening, anastomotic leakage, digestive, cutaneous or vascular fistulas, intra-abdominal hemorrhage), which has a high (66%).

For our patient, a total colectomy with ileo-rectal anastomosis was performed.

## 3 Conclusion

SEDV is a rare pathology, the diagnosis is difficult because of the ignorance of the pathology and the inaccessibility of genetic tests. However, it should be keeped in mind when there is any spontaneous colonic perforation in the young people.

The surgical management of colonic perforations recommends total colectomy with ileo-rectal anastomosis or definitive ileostomy as a preventive measure, justified by the high rate of recurrence and anastomotic fistulas.

## Ethical approval

The study is exempt for ethical approval.

## Sources of funding

None.

## Author contribution

**Khalid Elhattabi:** manage the research and design and write the first draft of the manuscript.

Hasna Benghait: manage the research and design and write the first draft of the manuscript.

**Abdelilah Elbakouri:** manage the research and design and write the first draft of the manuscript.

**Mounir Bouali:** manage the research and design and write the first draft of the manuscript.

**Fatimazahra Bensardi:** correction of the paper.

**Abdelaziz Fadil:** correction of the paper.

## Research registration

This is not the first case and the registration is not required.

## Guarantor

The Guarantor is the one or more people who accept full responsibility for the work and/or the conduct of the study, had access to the data, and controlled the decision to publish.

## Declaration of competing interest

The authors declare having no conflicts of interest for this article.
